# Effectiveness of two intramuscular combined vaccines for the control of *Mycoplasma hyopneumoniae* and porcine circovirus type 2 in growing pigs: a randomized field trial

**DOI:** 10.1186/s40813-021-00220-3

**Published:** 2021-06-27

**Authors:** Gwenaël Boulbria, Sophie Brilland, Charlotte Teixeira-Costa, Mathieu Brissonnier, Mathieu Charles, Nathalie Capdevielle, Valérie Normand, Franck Bouchet, Pauline Berton, Roman Krejci, Arnaud Lebret

**Affiliations:** 1 Porc.Spective Swine Vet Practice, ZA de Gohélève, 56920 Noyal-Pontivy, France; 2 rezoolution Pig Consulting Services, ZA de Gohélève, 56920 Noyal-Pontivy, France; 3 Ceva Animal Health, 10, avenue de la Ballastière, 33500 Libourne, France

**Keywords:** *Mycoplasma hyopneumoniae*, Porcine circovirus 2, Vaccine, qPCR, Serology

## Abstract

**Background:**

*Mycoplasma hyopneumoniae* and Porcine circovirus type 2 are two economically important pathogens affecting growing pigs. Control and prevention of both diseases can be accomplished by vaccination, together with biosecurity and good management practices. Many commercial vaccines are available. The aim of this study was to assess the efficacy of Hyogen® and Circovac® administered mixed at weaning and to compare this protocol with a competitor ready-to-use (RTU) vaccine.

**Case presentation:**

A randomised field trial was designed in a commercial farrow-to-finish farm located in France. A total of 641 pigs born from 54 different sows were included in this study. Piglets at weaning were allocated into three groups: the first one vaccinated with Hyogen® and Circovac® combined (group A), the second one vaccinated with a competitor RTU vaccine (group B) and the last one unvaccinated. Only minor local reactions for both vaccination groups could be observed which revealed a good safety of both protocols. Both vaccination schemes in this trial didn’t improve wean-to-slaughter growth performances but significantly reduced lung lesions, lung fissures and pleurisy at slaughter, produced a seroconversion for both *M. hyopneumoniae* and PCV-2 and significantly reduced the PCV-2 viral load in blood. When we compared groups A and B, we observed no significant differences in growth performances, mortality, clinical signs, percentages of affected lungs at slaughter, lung fissures and pleurisy, and no difference in pathogens detection. However, two statistical differences were observed between both vaccines: the mean lung lesion score and the percentage of extensive lung lesions were lower in group A. This is consistent with lower *M. hyopneumoniae* loads in the lower respiratory tract in pigs from group A but this difference was not statistically significant.

**Conclusions:**

Results reported in this case study must be considered with caution since it was done in only one farm. In this trial, Hyogen® and Circovac® mixed together under field conditions offered a successful protection of growing pigs and significantly decreased the extension of lung lesions during a natural field challenge when compared with a competitor RTU vaccine.

## Background

Porcine respiratory disease complex (PRDC) is a multifactorial disease caused by a combination of pathogens (bacteria and viruses). This term was used to describe the complexity of facts leading to the development of pneumonia, including pathogens but as well different factors such as environment, management practices. *Mycoplasma hyopneumoniae* (*M. hyopneumoniae*) is a bacteria known as the primary etiological agent of enzootic pneumonia (EP) which plays an important role in PRDC [16]. The infection is clinically characterized by a non-productive cough and causes cranio-ventral pulmonary consolidation lesions. The economic impact is huge due to a decrease in growth performance and an increase of feed conversion ratio and medication [9]. Despite all efforts performed and vaccination strategies set up to control this pathogen, *M. hyopneumoniae* continues to be an important concern for worldwide swine herds [16]. Porcine circovirus type 2 (PCV-2), a circular, single stranded, non-enveloped deoxyribonucleic acid (DNA) virus has been demonstrated to be present in almost all commercial swine herds. PCV-2 is the causative agent of the porcine circovirus-associated diseases which can have different clinical manifestations as PCV-2 systemic disease, historically known as post-weaning multisystemic wasting syndrome, PCV-2 reproductive disease, porcine dermatitis and nephropathy syndrome, and subclinical infection [26]. PCV-2 plays an important role in co-infections because the infection may downregulate the host immune system and enhance the infection and replication of other pathogens such as swine influenza viruses or porcine reproductive and respiratory syndrome virus [20]. Vaccination plays an important role in the control of *M. hyopneumoniae* and PCV-2. Inactivated and adjuvanted whole-cell vaccines are commonly used in commercial pig farms to control enzootic pneumonia [12]. However, protection against clinical signs is incomplete and vaccines do not totally prevent colonization [19]. For both pathogens, vaccine efficacy varies between herds, depending on infection level and infection dynamics within the herd and from farm management practices (biosecurity). Efficacy of PCV-2 commercial vaccines has been widely demonstrated on the limitation of PCV-2 systemic disease, reduction of PCV-2 viremia, shedding and improvement of clinical health status (decrease of co-infections and upgrade of production parameters) [25]. Several vaccines are nowadays available on the European market and different vaccine strategies can be implemented in farms to better manage the PRDC. The aim of the present study was to perform a trial to compare the efficacy of two one-shot commercial vaccines Hyogen® and Circovac®, respectively *M. hyopneumoniae* and PCV-2 vaccines, used mixed together (Ceva, Libourne, France) and a competitor one-shot bivalent ready-to-use (RTU) vaccine, against *M. hyopneumoniae* and PCV-2 field challenges.

## Case presentation

### Materials and methods

#### Herd selection

This field study was performed between October 2018 and May 2019 in a one site farrow-to-finish farm located in Brittany (France), in a high swine density area. This farm operated a three-week batch production system. The farm had a history of respiratory clinical signs in fattening pigs and EP-compatible lung lesions at slaughter. The herd was free from porcine respiratory and reproductive syndrome virus. In this farm, regarding the breeding herd, only gilts were vaccinated during quarantine against PCV-2 and *M. hyopneumoniae*. Piglets were vaccinated against both pathogens using a commercial ready-to-use combined vaccine. One month before the study, tracheobronchial swabs (TBS) and blood samples were collected from 4-week, 10-week, 16-week and 22-week-old-pigs (15 pigs per age). At least one pool per age was positive for *M. hyopneumoniae* on TBS using quantitative polymerase chain reaction (qPCR) on samples pooled by three. PCV-2 was not detected in blood using qPCR whatever the age. Lung lesions assessment was performed before the trial on 154 pigs. The percentage of pigs with EP-compatible lung lesions was around 40% at slaughter and a mean lung score of 2.3 was observed (the method was the same as the one used to evaluate lung lesions in the trial described below).

#### Study design

##### Type of trial

A randomized trial was carried out to compare the effectiveness of two one-shot commercial vaccines Hyogen® and Circovac® used mixed together with another ready-to-use bivalent vaccine available on the market.

##### Randomization and vaccination

A total of 641 pigs from 54 different sows were included in this study. The mean parity number of selected sows was 3.7. On average, 11.9 ([8–14]) piglets per sow were included. Early-weaned piglets were excluded. At 3 weeks of age, for each litter, all piglets were individually ear-tagged and randomly allocated in the following treatment groups.

Piglets in group A were vaccinated once intramuscularly with 2.5 mL of commercial vaccines mixed: an inactivated PCV-2a based vaccine with an oil/water adjuvant (Circovac®), mixed with an inactivated *M. hyopneumoniae* vaccine with oil adjuvant (Hyogen®). Briefly, Circovac® was re-constituted as per manufacturer’s instructions and then 50 mL of Circovac® were mixed with 200 mL of Hyogen® in a sterile container. Piglets in group B were vaccinated once intramuscularly with 2 mL of a commercial ready-to-use bivalent vaccine. For both groups, piglets were injected at the left side of the neck. Piglets from each sow were randomly assigned to these two treatments, and randomization was performed using the Excel RAND function (Excel 2016, Microsoft Corporation, USA) with a 1:1 allocation ratio, except 20 pigs, randomly selected, which were not vaccinated and constituted the control group (Group C). The piglets were visually observed for immediate reactions during or immediately after vaccination and general health at vaccination and 1 h after vaccination.

All pigs were mixed in the same rooms and same pens during the study, so that all three treatments were represented in each pen, in both nursery (30 pigs per pen) and finishing barns (15 pigs per pen). They were subjected to the same management practices. Pigs were fed with on-farm feed.

#### Clinical and performance parameters

Pigs were observed daily by the farmer for clinical disease or death. All individual treatments were recorded. Coughing was assessed every 3 weeks by four of the authors simultaneously in order to discriminate the treatment group (A or B) based on the color of ear tags. Briefly, non-productive dry coughs were counted in each room for 2 min (after pigs had been encouraged to move and their activity had gone back to normal). A respiratory disease score (RDS) were expressed as a number of coughs per 100 pigs per 2 min for each group [1]. Measurements were performed at 3, 6, 9, 12, 15, 18, 21 and 24 weeks of age (woa). Clinical signs were not recorded in control pigs.

All pigs were individually weighed at inclusion and at slaughter to determine average daily gain (ADG) (g/pig/d) during the study period, by subtracting the inclusion weights from the slaughter weights divided by the number of days during the respective periods.

#### Post-mortem examination

##### Necropsy

When mortality occurred, pigs were necropsied for macroscopic observation of lungs lesions and PCV-2-like lesions. If gross lesions compatible with *M. hyopneumoniae* and/or PCV-2 were present, samples were submitted for laboratory analysis. For lungs with EP-compatible lesions, a *M. hyopneumoniae* qPCR on lungs was performed. For organs with PCV-2-compatible lesions, a PCV-2 qPCR and a histopathological observation plus detection of PCV-2 in tissues should be performed.

##### *M. hyopneumoniae* lung lesion scoring

Extension of EP-compatible lung lesion and presence of fissures and pleurisy were recorded at slaughter by the first author before the sanitary inspection. EP-compatible lesions were defined as red-purplish areas of cranioventral pulmonary consolidation with a liver-like consistency [1]. A 24 points scale was used for macroscopic lung lesions [11]. Briefly, excepted azygos lobe, each lobe was individually assessed by visual estimation of the proportion of lung with EP-compatible lung lesion, and scored between 0 (absence of lesion) to 4 (lesion over 75% of the lobe surface). Points per lobe were summed to provide an overall area lung score.

Chronic EP-compatible lesions (fissures) were grey to purplish cranioventral scars, shrunken below the surface of the lobes with a more solid texture than unaffected parenchyma [1]. Pleurisy was evident fibrous adhesion between lung lobe(s) and other lung lobe(s) or thoracic wall [1]. For each pig, pleurisy was scored for cranial pleurisy between 0 and 1 (absence or presence of pleurisy) and for caudal pleurisy between 0 (absence of lesion) and 4 (severely extended bilateral lesion, at least 1/3 of both diaphragmatic lobes) [18].

#### Samples

Samples were collected individually from the same 30 randomly selected pigs from group A, 30 pigs from group B and 20 pigs from group C every 3 weeks (at 3, 6, 9, 12, 15, 18, 21 and 24 weeks of age).

##### Blood samples

Blood was collected by venipuncture (jugular vein) in Vacutest® tubes and submitted to the laboratory within 12 h under positive-cold conditions.

##### Tracheobronchial swabs

Pig’s mouth was held open with a gag. TBS were collected by deep insertion into the trachea of a sterile catheter (Euromedis, Neuilly-sous-Clermont, France), rotated and moved up-and-down. The extremity of the catheter was cut into a sterile tube containing 1 mL of Buffered Peptone Water Broth and stored under positive-cold conditions.

#### Detection and quantification of *M. hyopneumoniae* DNA

Tracheo-bronchial swabs were vortexed and then centrifugated (12.000 g, 20 min) and the pellets were resuspended in 800 μL of lysis solution. DNA was extracted from 200 μL EDTA blood samples using MagAttract 96 Cador Pathogen kit (Qiagen, Venlo, The Netherlands) following manufacturer’s instructions. Finally, DNA recovery was obtained in 100 μL elution buffer AVE. *M. hyopneumoniae* detection was achieved using a qPCR test previously described [17]. Dilutions were used for absolute quantification assays. Samples with a Ct lower than 40 and curve showing specific exponential shape were considered as positive.

#### Detection of antibodies against *M. hyopneumoniae*

Detection of antibodies against *M. hyopneumoniae* in serum was tested with the commercial HerdChek *M. hyopneumoniae* enzyme-linked immunosorbent assays (ELISA) (IDEXX Laboratories, Westbrook, Maine, USA), based on the optical density (OD) value of the sample. Results were expressed as S:P ratio, defined as (sample OD-negative control OD) ÷ (positive control OD – negative control OD). Sample-to-positive ratios ≥0.4 were considered positive, S: P ratios < 0.4 but ≥0.3 were classified as suspect, and S: P ratios < 0.3 were classified as negative. Results were also presented considering samples with S:P ratio > 1.5.

#### Detection and quantification of PCV-2 DNA

For detection and quantification of PCV-2 DNA, blood samples were pooled by five by mixing and vortexing five individual 100 μL blood samples. The total DNA was extracted from sera using QIAamp DNA miniKit (Qiagen, Venlo, The Netherlands) following the manufacturer’s protocol. One hundred microliters of sample were added to 180 μl of lysis buffer + proteinase K and incubated for 1 h à 56 °C. At the end of the protocol, total DNA was eluted in 200 μl of AE buffer and kept at − 20 °C until use. PCV-2 was detected and quantified using VetMaxTM PCV-2 Quant Kit (Thermo Fisher Scientific, Hampshire, UK) following manufacturer’s instructions. For each assay, positive and negative controls were tested with field samples. Samples with a Ct lower than 40 and curve showing specific exponential shape were considered as positive.

#### Detection of antibodies against PCV-2

Presence of antibodies against PCV-2 in blood were tested with the commercial ELISA Ingezim Circo IgG 11. PCV.K1 kit (Eurofins Ingenasa, Madrid, Spain) following manufacturer’s instructions. Results were expressed as mean S:P ratio values (S = sample OD; P = mean positive control OD).

#### Statistical analysis

Clinical data, performances and laboratory results were collected into a database (Office Excel 2019, Microsoft, Redmond, USA). qPCR data were log10 transformed. Statistical analyses were carried out using R Studio version 4.0.2 (R Core Team 2020). The level of significance was set at *p* < 0.05. In each analysis, comparison between the three groups was done. Analysis of quantitative variables was performed using a non-parametric Kruskal-Wallis test. These variables were body weights, ADG, mortality, RDS, lung lesion score, pleurisy scores, the number of *M. hyopneumoniae* copies in samples and areas under the curves. S:P ratios per age for both *M. hyopneumoniae* and PCV-2 ELISAs were compared using an ANOVA. For categorical variables (percentage of pigs with lung lesions and percentage of positive pigs with laboratory tests) the Fisher-test was used.

## Results

Clinical and local injection site reactions observations, immediately after vaccination and 1 h later, revealed a good safety of both protocols. Only minor local reactions for both vaccination groups could be observed.

Performance parameters were recorded and lung lesion were evaluated for 273 pigs from group A, 269 pigs from group B and 15 pigs from group C. At the end of the trial, before first slaughtering, laboratory results at each sample points were obtained from 29 pigs from group A, 28 from group B and 18 from group C. Pigs lacking at the end of the trial died or had lost their ear tags or had unreadable ear tags before examination in the slaughterhouse.

### Performance parameters

Performance parameters are presented in Table 1. No statistically significant difference for the average body-weight were observed between groups neither at inclusion (3 weeks of age) nor before slaughter. Average ages at slaughter were similar in the three groups: pigs were aged of 174.9 days, 175 days and 175.1 days in group A, B and C respectively. No statistically significant differences were shown for ADG from inclusion to slaughter between all treatment groups. A total of 13 pigs (309 included, 4.2%) and 16 pigs (312 included, 5.1%) died in group A and B respectively during the study. We couldn’t compare with mortality in group C because of ear tags lost during the trial. Euthanasia of weak pigs and digestive syndrome, including colibacillosis in nursery pigs and haemorhagic bowel syndrome in fattening pigs, were the three causes of mortality. At the end of the study, no statistically significant difference in mortality rates were observed among group A and group B.

**Table 1:** Performance parameters in the three groups (A and B were vaccinated, C was unvaccinated). Different letters in superscript within a line means *p* < 0.05 (Kruskal-Wallis test)

	Group A	Group B	Group C
Performance parameters	*n* = 273	*n* = 269	*n* = 15
Average bodyweight (kg) at inclusion	5.26^a^	5.23^a^	5.7^a^
Average bodyweight (kg) at slaughter	119.2^a^	119.9^a^	119.9^a^
Average age at slaughter (days)	174.9^a^	175^a^	175.1^a^
ADG (g/d)	723.3^a^	727.9^a^	727.5^a^
Mortality	4.2%^a^	5.1%^a^	

### Clinical signs

**Fig. 1:**
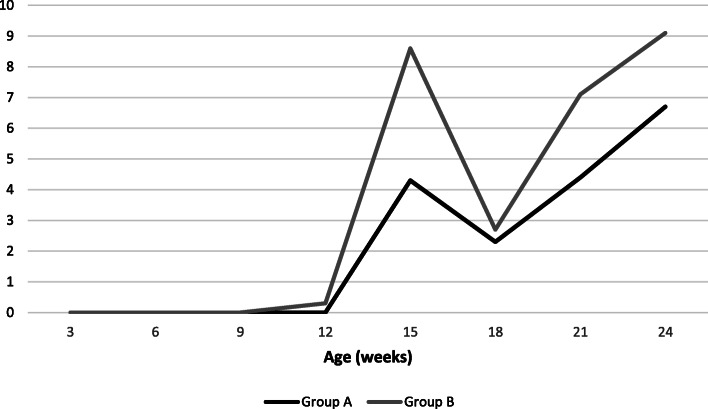
Average respiratory disease score (RDS) during the trial in group A and group B

No clinical sign related to PCV-2 systemic-disease were observed during the course of the study. Respiratory clinical signs were recorded in treatment groups. The number of pigs that coughed increased during the study in both groups. An increase in RDS was recorded from 15 woa with a higher RDS in group B compared with group A at each sampling time (Fig. 1). However, there was no significant difference between both groups.

### Macroscopic lung lesions

The results of macroscopic lung evaluation are summarized in Table 2. Lung and thoracic wall condemnations were performed for one pig in group A and three pigs in group B by sanitary inspector at slaughterhouse. 46, 51 and 85% of the lungs had macroscopic visible EP-compatible lesions in group A, B and C respectively. 10, 13 and 65% of the lungs had visible fissures in group A, B and C respectively. Percentages of pigs with macroscopic lung lesions and with fissures were lower in group A compared with group B, but this difference was not statistically significant. The difference was significant between vaccinated and unvaccinated groups. No difference was observed in the percentage of pigs with pleurisy between group A and group B. For these three criteria, a statistically significant difference was observed between group C (non-vaccinated) and vaccinated groups (A and B). Regarding the mean lung lesion score, a statistically significant difference was shown between the three groups. We observed a lower extension in lung lesions in group A compared with group B. Mean lung lesion score in group C was substantially higher than in group A and B. Moreover, the percentage of pigs with extensive EP-compatible lesions, defined as lung lesion score ≥ 6, was significantly lower in group A compared with group B.

**Table 2:** Macroscopic lung lesions evaluated at slaughter age. Different letters in superscript within a line means *p* < 0.05

	Group A ***n*** = 273	Group B ***n*** = 269	Group C ***n*** = 15
Respiratory condemnations at slaughter	1	3	0
Percentage of EP-compatible lesions *	46%^a^	51%^a^	85%^b^
Mean lung lesion score **	3.2^a^	4.2^b^	11^c^
Percentage of pigs with extensive EP-compatible lesions*	35.4%^a^	45.9%^b^	66.7%^c^
Percentage of pigs with lung fissures *	10%^a^	13%^a^	65%^b^
Percentage of pigs with pleurisy *	1.5%^a^	1.5^a^	30%^b^
Mean pleurisy score **	1.8^a^	2^a^	2.5^b^

* These criteria were statistically compared using Fischer test

** These criteria were statistically compared using Kruskal-Wallis test

### *M. hyopneumoniae* results

#### Detection and quantification of *M. hyopneumoniae* DNA

**Fig. 2:**
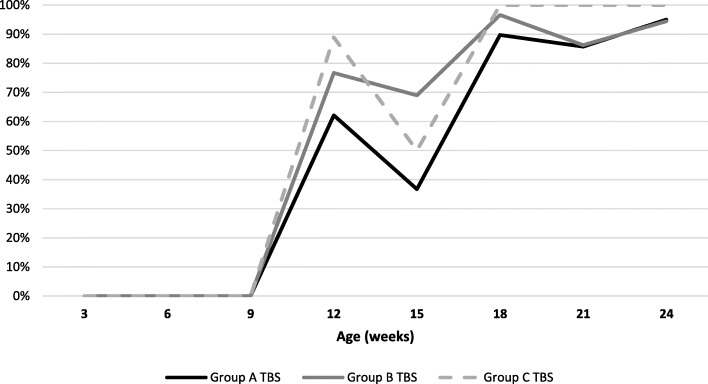
*M. hyopneumoniae* qPCR results expressed in percentage of positive tracheobronchial swabs per treatment group and sampling point

All pigs sampled during the study remained qPCR negative until 9 weeks of age. *M. hyopneumoniae* was firstly detected at 12 weeks of age in all groups (Fig. 2). This confirmed that pigs were challenge with *M. hyopneumoniae* during the trial. A higher percentage of qPCR positive pigs on TBS sample was observed in group C (non-vaccinated) and group B compared with group A at 12 woa (88.9% for group C, 76.7% for group B and 62.1% for group A), at 15 woa (50% for group C, 69% for group B and 36.7% for group A), at 18 woa (100% for group C, 96.6% for group B and 89.7% for group A) (Fig. 2). These differences, however, were not statistically significant.

**Fig. 3:**
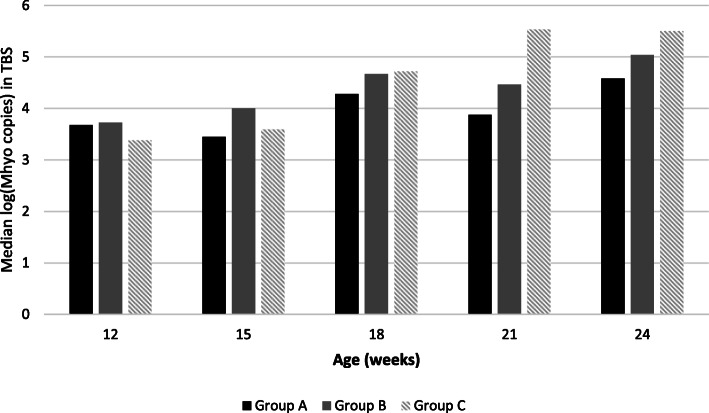
Median *M. hyopneumoniae* copies/ml in qPCR positive samples (expressed in logarithmic scale) per treatment group and sampling point

*M. hyopneumoniae* bacterial load within qPCR positive tracheobronchial swabs ranged from 1.6 × 10^2^ fg/mL to 5.6 × 10^8^ fg/mL. Median number of log copies of *M. hyopneumoniae* detected in each group are presented in Fig. 3. A lower *M. hyopneumoniae* load in TBS was observed in group A compared with group B and group C. However, no statistically significant difference was found between each treatment group at any sampling point.

#### Detection of antibodies against *M. hyopneumoniae*

**Fig. 4:**
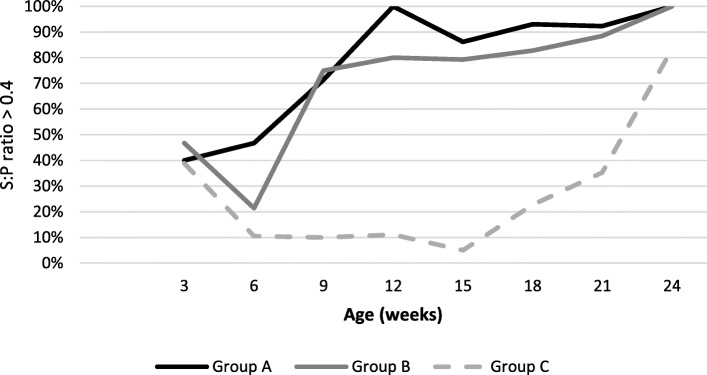
Percentage of *M. hyopneumoniae* seropositive pigs per treatment group and sampling age using Idexx HerdChek *M. hyopneumoniae* ELISA. In this graph, seropositivity is defined as S:P ratio > 0.4

**Fig. 5:**
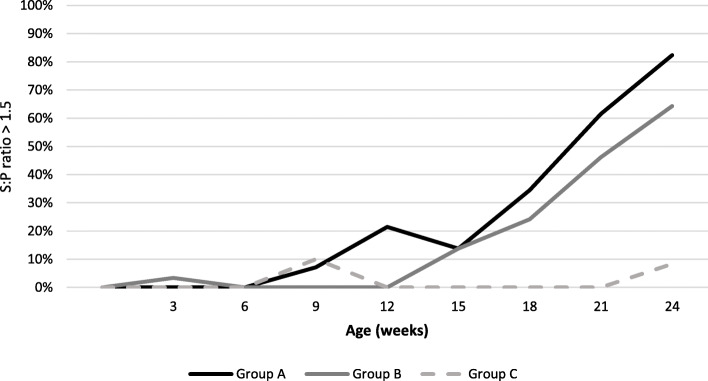
Percentage of *M. hyopneumoniae* pigs with S:P ratio > 1.5 per treatment group and sampling age using Idexx HerdChek *M. hyopneumoniae* ELISA

Percentage of *M. hyopneumoniae* seropositive animals are represented in Fig. 4 based on the threshold of 0.4 for S:P ratio and in Fig. 5 based on the threshold of 1.5 in S:P ratio.

Using the threshold in S:P ratio of 0.4, we observed ELISA positive pigs in all groups during the trial. Around 40% of piglets were seropositive at inclusion (weaning) in all groups. We observed a decrease in number of positive between 3 woa and 6 woa in group C (non-vaccinated) and group B, and a major increase between 6 woa and 9 woa in group A and B (vaccinated). During the study, at least 20% of vaccinated pigs were ELISA positive whatever the sampling time. From 12 woa until the end of the study, more than 80% pigs were seropositive in vaccinated pigs. An increase in percentage of positive pigs in group C was observed from 15 woa. No statistical significant difference was observed between group A and group B in the percentage of seropositive pigs at each sampling time. But a statistically significant difference was observed between vaccinated groups (A and B) and non-vaccinated group (C) when comparing the areas under the curve (Fig. 4).

We observed an increase in the percentage of pigs with a S:*P* value > 1.5 in vaccinated pigs from 9 woa in group A and from 15 woa in group B. From 15 woa, percentage of pigs with a S:P value > 1.5 in vaccinated pigs increased quickly up to more than 60% at the end of the study. The percentage of pigs with a S:P value > 1.5 in group C (unvaccinated) remained under 10% during the trial. No statistical significant difference was observed between group A and group B in the percentage of seropositive pigs at each sampling time. But a statistically significant difference was observed between vaccinated groups (A and B) and non-vaccinated group (C) when comparing the areas under the curve (Fig. 5).

**Fig. 6:**
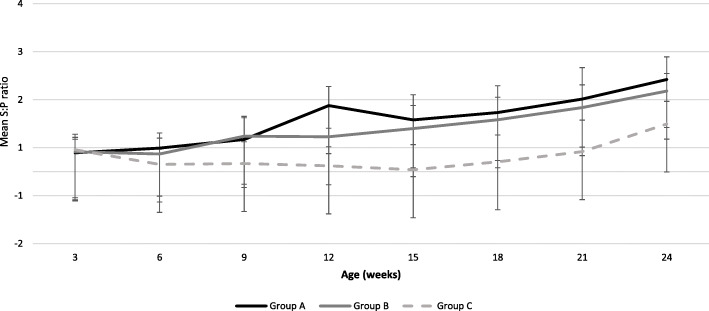
Mean (±SD) S:*P* values per treatment group and sampling age using Idexx HerdChek *M. hyopneumoniae* ELISA

In the group C (unvaccinated pigs), significantly lower S:*P* values were detected, thus lower *M. hyopneumoniae* specific antibodies were detected compared to group A and group B (vaccinated pigs) (Fig. 6). We observed an increase in S:P values through the study. Between group A and group B, no statistically significant difference was observed in S:P values at each sampling time. But a statistically significant difference was observed between vaccinated groups (A and B) and non-vaccinated group (C) when comparing the areas under the curve.

### PCV-2 results

#### *Detection and quantification of* PCV-2 *DNA*

Four pools of sera were qPCR positive for PCV-2 DNA during the study confirming that pigs were exposed to a field challenge. One pool was positive in the group C at 18 woa, with a viral load in pooled sera of 6.2 × 10^7^ copies per mL. At 24 woa, two pools were positive in the group C (one pool with a viral load lower than 10^4^ copies per mL (limit of quantification) and one pool with 4.5 × 10^5^ copies per mL). One pool in group A was also positive but below the limit of quantification at this age.

#### *Detection of antibodies against* PCV-2

**Fig. 7:**
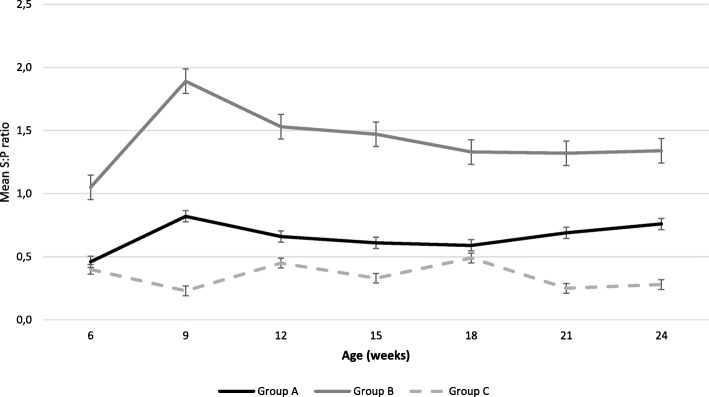
Mean (±SD) PCV-2 S:P ratio ELISA values per treatment group and sampling age using Ingezim Circo IgG 11. PCV.K1 kit

Mean S:P ratio ELISA values per treatment group and per sampling age are presented in Fig. 7. Unfortunately, data at the time of inclusion and vaccination are not available because of insufficient quantity of sera. From 6 weeks of age until the end of the study, pigs from group B showed a significantly higher S:P value than pigs from groups A and C. Moreover, pigs from group A showed higher S:P value than pigs from group C whatever the sampling point.

## Discussion

*M. hyopneumoniae* commercial vaccines are widely applied [15] and PCV-2 vaccines are currently the most sold preventive products in swine worldwide [25]. Both valences could be associated in combined vaccination strategies for growing pigs in order to decrease labour expenses and to improve welfare and health by decreasing the number of injection and manipulations. The aim of this trial was to assess the efficacy of Hyogen® and Circovac® (Ceva Animal Health, Libourne, France) applied combined during a field challenge with *M. hyopneumoniae* and PCV-2 compared with an available ready-to-use vaccine in growing pigs. Based on our results, the effectiveness of Hyogen® and Circovac® mixed was concluded.

In this study, after randomization, growing pigs from the two vaccine groups and the control group were mixed together in order to homogenize as much as possible environmental, feeding and management conditions and exposure to pathogens. However, a bias in vaccine effectiveness could be introduced with this method. The reduction in pathogens shedding (*M. hyopneumoniae* and PCV-2 in our trial) after vaccination of pigs of one vaccine group could contribute to minimize the infectious pressure of the pigs of the other groups reared in the same environment.

This study was designed to determine the effectiveness of Hyogen® and Circovac® mixed together compared with a ready-to-use vaccine on growth, mortality, respiratory clinical signs, macroscopic lung lesions, *M. hyopneumoniae* detection in lower respiratory tract, PCV-2 detection in blood and serologic profile for the three groups for both pathogens.

The major advantages of *M. hyopneumoniae* vaccination include improvement of daily weight gain (2–8%) and shorter time to reach slaughter weight, improvement of feed conversion ratio (2–5%), reduced clinical signs and sometimes mortality rate [13, 14]. Similarly, both experimental and field studies have demonstrated that PCV-2 vaccination in piglets is very efficient [25]. PCV-2 commercial vaccines available to date show a remarkable efficacy with a reduction in PCV-2-associated production losses in the growing-finishing stage [25]: improvement of average daily weight gain, and feed conversion rate, a decreased in PCV-2 systemic disease occurrence and clinical severity, and a reduced mortality rate. In our study, we observed no statistical difference in average body weight and age at slaughter, average daily weight gain between the three groups. As the three groups were mixed together during the trial, and because of a small number of pigs constituting the control group, the absence of degradation of growth performances in unvaccinated pigs could be explained by a decrease of the infectious pressure at the herd level due to the presence of a large population of vaccinated pigs. We observed also no significant difference in mortality rate between both vaccinated groups. Moreover, no statistical difference was noticed in respiratory clinical signs intensity between both vaccine groups and also no clinical signs related to PCV-2 systemic disease were observed.

Percentage of lung lesion reduction in vaccinated pigs is one of the main parameters used to measure *M. hyopneumoniae* vaccine efficacy, since *M. hyopneumoniae* was considered one of the most important primary bacterial respiratory pathogens associated with such lung lesions [7]. Keeping in mind that EP-compatible lesions are non-pathognomonic of *M. hyopneumoniae* infection as other respiratory diseases can produce similar lesions [30]. The scoring method is based on visual and manual assessment of the affected proportion of the lung and might be considered subjective. In this study we scored lungs using French scoring scale [11] because of experience of the first author in the use of this method. A high correlation between lung lesions scoring systems were observed in a previous study [8]. Mean lung lesion scores were higher during the trial than at herd selection due to H1avN1 influenza outbreak during the trial. This outbreak occurred in finishing pigs. In our study we observed a remarkable reduction in the percentage of lung with EP-compatible lesions, in the average lung lesion score, and in the percentage of pigs with fissures and pleurisy between vaccinated and unvaccinated pigs. This data confirmed the effectiveness of both vaccines in prevention of *M. hyopneumoniae* lung lesions, reducing the number of pigs showing macroscopic lung lesions as well as their extension. Moreover, we observed that pigs from group A had a significant reduction in the mean lung lesions score and in the percentage of extensive lesions. However, as noticed previously, we observed no difference in performance data between groups. Previous studies indicated that we could observe a negative correlation between pneumonia scores and performance data such as growth and age at slaughtering [22]. We can also suppose that the number of pigs included in our study was not sufficient to observe a statistical difference in performance data between groups because of the trial design.

In this study we detected *M. hyopneumoniae* in trachea-bronchial swabs of all groups from 12 weeks of age. Detection of the pathogen in all treatment groups confirmed that *M. hyopneumoniae* vaccination did not prevent from the infection [14]. Vaccination was related with a lower *M. hyopneumoniae* prevalence at slaughter age in upper respiratory airways (nasal cavities and tonsils) as previously described [3, 24, 28]. In our study we did not observe any statistical difference between the three groups in *M. hyopneumoniae* load in the lower respiratory tract, but, from 21 weeks of age, mean bacterial loads were higher in unvaccinated pigs. This data could be linked with the higher severity and extension of lung lesions in unvaccinated pigs as previously described. The combination of both observations confirmed the effectiveness of both vaccines in preventing *M. hyopneumoniae* infection and disease. This is in accordance with previous study reported that the protection against clinical pneumonia is often incomplete and vaccines do not prevent colonization [29], but indicate that the currently used vaccines may reduce the number of organisms in the respiratory tract [19] and may decrease the infection at the herd level [28]. The high prevalence of *M. hyopneumoniae* in trachea-bronchial swabs in pigs from all groups in our study suggested that natural infection occurred during our trial [5].

RDS alone cannot be used as a solely indicator of *M. hyopneumoniae* disease, as cough is not a pathognomonic sign of infection. The typical dry cough that accompanies EP does not develop immediately after infection, at least 6 days seems to be a minimum for a cough to be heard in inoculated animals [2]. But this period can vary greatly under field conditions [15]. In our study we detected *M. hyopneumoniae* first at 12 weeks of age. Cough was heard from 12 weeks of age in group B (with a weak percentage of pigs heard coughing) and from 15 weeks of age in group A. This data confirmed the delay between infection and development of dry cough. RDS increased in line with *M. hyopneumoniae* bacterial loads in the lower respiratory tract and the seroprevalence increase during the trial.

Antibodies can be the result of natural *M. hyopneumoniae* infection, maternal antibody absorption in piglets [4] or can be generated after vaccination [15]. Unfortunately, antibodies against *M. hyopneumoniae* are detected with ELISA tests regardless of origin, which complicates the interpretation of serological results in practice. Considering a S:P ratio threshold of 0.4, the percentages of seropositive pigs in both vaccinated groups were similar and reached more than 80% from 12 woa. At this stage, we observed no seroconversion in unvaccinated pigs. The percentage of seropositive pigs increased from 18 woa in unvaccinated pigs, with a remarkable increase just before slaughter. However, *M. hyopneumoniae* infection was detectable from 12 woa. This is in accordance with previous studies which reported that vaccination elicits a detectable humoral response [28] and that natural circulating antibodies are developed several weeks after infection [10, 23]. Because of the inability of ELISA tests to differentiate natural infection from vaccination, the threshold of 1.5 have been proposed in practice to differentiate vaccinated pigs that suffer from EP, assuming that vaccination plus natural exposure would have a higher serologic response [6]. Considering this threshold, we observed a remarkable increase in circulating antibodies from 12 woa in group A and 15 woa in group B, closer with the detection of *M. hyopneumoniae* in trachea-bronchial swabs. These results supported the usefulness of this threshold for *M. hyopneumoniae* serological monitoring in practice in vaccinated pigs. In a previous study, vaccination against *M. hyopneumoniae* resulted in a significantly higher percentage of seropositive animals 3 weeks after vaccination [27], whereas in our trial a significant increase in the percentage of seropositive pigs was observed 6 weeks after vaccination. In both vaccinated groups, mean S:*P* values were similar at all sampling times, but no correlation between antibody titers and protection against the infection is demonstrated to date.

We suspected that pigs were challenged with PCV-2 during the trial because we detected PCV-2 DNA in unvaccinated pigs at the end of fattening with one pool which had a viral load higher than 10^7^ copies per mL. PCV-2 vaccines protect by reducing the amount of PCV-2 present in the pigs [21]. Our results are consistent with this effect of both vaccines since PCV-2 DNA was significantly detectable only in unvaccinated pigs. No clinical sign consistent with PCV-2-AD was observed in the three groups during the study. Both groups vaccinated against PCV-2 showed a significantly higher S:P value than unvaccinated group. Moreover, we showed a significant difference in S:P values between both vaccinated groups. The reason for this observation is unknown but could be explained by the intrinsic composition of both PCV-2 vaccines compared in our study. Means S:P ratios measured during the trial in pigs vaccinated with Hyogen® and Circovac® mixed were in accordance with one previously published study after inoculation, S:P ratios varied between 0.2 and 0.8 [27]. We detected PCV-2 DNA in blood from unvaccinated pigs from 18 weeks of age but we didn’t observe seroconversion between 18 weeks of age and the end of the trial.

## Conclusion

These results improve the knowledge about the effectiveness of Hyogen® and Circovac® mixed together. Keeping in mind that the percentage of unvaccinated pigs in the population studied was small, vaccination of pigs didn’t improve growth performances but significantly reduced EP-compatible lesions, lung fissures and pleurisy at slaughter, produced a seroconversion for both *M. hyopneumoniae* and PCV-2 and significantly reduced the PCV-2 viral load in blood. However, we observed no difference in the percentages of *M. hyopneumoniae* positive pigs using qPCR but, even if we couldn’t show a statistical difference, the *M. hyopneumoniae* loads in lower respiratory tract were lower in vaccinated pigs. Moreover, these results demonstrated the effectiveness of Hyogen® and Circovac® mixed when compared with a competitor RTU vaccine. Indeed, we observed no difference in growth performances, mortality, clinical signs, percentages of EP-compatible lesions at slaughter, lung fissures and pleurisy, and no difference in pathogens detection. However, three statistical differences were observed between both vaccination schemes: the mean lung lesion score and the percentage of extensive EP-compatible lesions and mean PCV-2 ELISA S:P ratio were reduced. However, these results must be considered with caution and need further investigations to be confirmed because this trial was performed in only one farm.

## Data Availability

For this randomized controlled trial, this manuscript was written according to the checklist CONSORT. All datasets used in this study are available from the corresponding author on reasonable request.

## Abbreviations

ADG: Average Daily Gain
ANOVA: ANalysis Of VAriance
DNA: Deoxyribonucleic acid
ELISA: Enzyme-Linked Immunosorbent Assays
EP: Enzootic Pneumonia
*M. hyopneumoniae*: *Mycoplasma hyopneumoniae*
OD: Optical Density
PCV-2: Porcine circovirus type 2
PRDC: Porcine Respiratory Disease Complex
qPCR: Quantitative Polymerase Chain Reaction
RDS: Respiratory Disease Score
RTU: Ready-To-Use
TBS: Tracheo-Bronchial Swabs
WOA: Weeks Of Age
